# eduSPIM: Light Sheet Microscopy in the Museum

**DOI:** 10.1371/journal.pone.0161402

**Published:** 2016-08-25

**Authors:** Wiebke Jahr, Benjamin Schmid, Michael Weber, Jan Huisken

**Affiliations:** 1 Max Planck Institute of Molecular Cell Biology and Genetics, 01307 Dresden, Germany; 2 Optical Imaging Centre Erlangen, Friedrich-Alexander-University of Erlangen-Nuremberg, 91054 Erlangen, Germany; 3 Harvard Medical School, Boston, Massachusetts 02115, United States of America; 4 Morgridge Institute for Research, Madison, Wisconsin 53715, United States of America; Imperial College London, UNITED KINGDOM

## Abstract

**Light Sheet Microscopy in the Museum:**

Light sheet microscopy (or selective plane illumination microscopy) is an important imaging technique in the life sciences. At the same time, this technique is also ideally suited for community outreach projects, because it produces visually appealing, highly dynamic images of living organisms and its working principle can be understood with basic optics knowledge. Still, the underlying concepts are widely unknown to the non-scientific public. On the occasion of the UNESCO International Year of Light, a technical museum in Dresden, Germany, launched a special, interactive exhibition. We built a fully functional, educational selective plane illumination microscope (eduSPIM) to demonstrate how developments in microscopy promote discoveries in biology.

**Design Principles of an Educational Light Sheet Microscope:**

To maximize educational impact, we radically reduced a standard light sheet microscope to its essential components without compromising functionality and incorporated stringent safety concepts beyond those needed in the lab. Our eduSPIM system features one illumination and one detection path and a sealed sample chamber. We image fixed zebrafish embryos with fluorescent vasculature, because the structure is meaningful to laymen and visualises the optical principles of light sheet microscopy. Via a simplified interface, visitors acquire fluorescence and transmission data simultaneously.

**The eduSPIM Design Is Tailored Easily to Fit Numerous Applications:**

The universal concepts presented here may also apply to other scientific approaches that are communicated to laymen in interactive settings. The specific eduSPIM design is adapted easily for various outreach and teaching activities. eduSPIM may even prove useful for labs needing a simple SPIM. A detailed parts list and schematics to rebuild eduSPIM are provided.

## Introduction

Science outreach programs are important to communicate the benefits of scientific research to the public. A successful outreach project captures people’s attention, be it through surprising findings or attractive data visualisation. In interactive settings, laymen can explore the research area according to their own agenda and interest. For this reason, modern exhibitions in technical and scientific museums are interactive.

The UNESCO declared 2015 as the International Year of Light and Light-Based Technologies with the goal to “raise awareness of how optical technologies […] provide solutions to worldwide challenges” [1]. In this context, the *Technische Sammlungen Dresden* (TSD, [2]), a museum dedicated to science education and technological history, together with partners from research, industry and culture, initiated a Dresden Year of Light. One of its main pillars is the special exhibition “Hi Lights!” in the TSD, where local institutes and companies showcase their work to the general public. Within this framework, we designed and built an educational fluorescence light sheet microscope to demonstrate how advances in technology drive discoveries in the life sciences.

Fluorescence light sheet microscopy (or Selective Plane Illumination Microscopy, SPIM, [3]) has become the technique of choice to image developing samples since its development more than a decade ago, [4, 5]. In addition to its popularity in science, light sheet microscopy is also ideal for teaching purposes: The setups can be kept fairly simple and most of the optics can be understood with basic optics knowledge. Since light sheet microscopy is mainly used to image larger objects in the mm range, such as entire embryos, the sample can be discerned by eye, which further adds to the descriptiveness of the microscope.

In a light sheet fluorescence microscope, illumination and detection are separated into two distinct optical paths that are perpendicular to each other. The sample is positioned at the intersection of both paths and illuminated with a thin sheet of light. Three dimensional (3D) data is acquired plane-wise while the sample is moved through the light sheet. In contrast to other fluorescence microscopy techniques, there is no out-of-focus fluorescence signal. Essentially, in SPIM optical sectioning is performed in the illumination path, while confocal microscopes suppress out-of-focus signal with a pinhole in the detection path. Phototoxicity is thus minimized in two ways in light sheet microscopy when compared to confocal techniques: Firstly, only the imaged sample regions are illuminated. Secondly, the detection arm is equipped with a fast camera to acquire widefield image data. Since this widefield detection scheme is highly parallelized, each camera pixel integrates much longer than the point detector in a confocal microscope at the same overall imaging speed. Therefore, the necessary illumination intensities are greatly reduced [6].

For the museum, the microscope had to meet specific requirements on hardware, software and sample preparation. Most importantly, the microscope had to fulfill its educational mission but at the same time had to be designed sturdy enough to “survive” at least one year in the museum. While simple and open designs existed for light sheet microscopes (OpenSPIM [7] and OpenSPIN [8]), these have been developed for scientific use and neither seemed suitable as museum exhibit.

In this paper, we present eduSPIM, a fully functional educational SPIM designed for a museum. Following the TSD’s concept, eduSPIM was conceptualized as a hands-on educational exhibit and invites the visitors to explore both the underlying optics of SPIM and the sample geometry. Users can navigate through the sample and decide autonomously which regions to investigate more thoroughly. The software controlling the microscope was designed to anticipate all potential error source and to allow for maximum flexibility on the user’s side. The eduSPIM layout and sample choice did not presume any prior knowledge from the visitors. We chose a fixed, durable sample and kept photobleaching minimal with an intelligent illumination concept only exposing the sample to light when needed. The eduSPIM’s beampath was designed to be self-explanatory and easy to understand. Overall, our eduSPIM was specifically built for a museum exhibition, but it is just as well suited for other teaching activities, e.g. in schools or universities. Moreover, since it is fully functional, the eduSPIM design could also be used in a lab where a simple, easy to operate light sheet microscope is needed.

## Design strategy for the eduSPIM

A microscope design for a museum is challenged by specific requirements on hardware, software and sample preparation that differ from the requirements for an instrument with a scientific purpose. For eduSPIM, space limitations in the museum, duration of the exhibition and untrained users demanded a small, robust and safe microscope, that should additionally be fully functional, fun to use and easy to understand. In this section, we compare the requirements placed on a research microscope to those placed specifically on eduSPIM and present the solutions we found to fulfill each demand.

Software is only discussed briefly, since any previously written control software could in principal be adapted to run eduSPIM. For operation in the museum however, downtime and maintenance of the setup had to be minimized. We found that a software framework developed for the control of scientific instruments in remote locations [9] was easily expanded to handle eduSPIM’s specific hardware components.

### Minimal user interface

A research microscope is usually constructed to perform a range of imaging tasks in various samples. To this end, parameters can be fed into a software interface and are adjusted flexibly in the microscope’s hardware. For eduSPIM, we restricted flexibility by defining both the sample and the imaging task beforehand and designed a simplified, clean hardware interface consisting of seven push buttons (input) labelled with pictograms and a standard computer monitor (output). With four push buttons, visitors moved the sample through the light sheet while viewing it on the screen. With the remaining three buttons, visitors acquired and displayed a 3D-stack, turned on the laser to visualise the beampath and displayed additional information on screen. Any other parameters, such as laser power, acquisition time and bounding box remained invisible to the public and were adjusted only during maintenance, e.g. when a fresh sample had been inserted.

### Imaging modalities and visualisation

In research environments, microscopy data is evaluated by trained scientists or technicians. Ideally, only a subset of cells or cellular components are fluorescently labelled. However, if the fluorescent signal is too sparse, data interpretation might be difficult even for experienced users. A fluorescent counterstain may be added to aid interpretation of the primary stain, but this approach requires a second laser line for excitation and is deleterious to sample longevity due to increased rates of photobleaching. To aid inexperienced visitors in the museum in interpreting the fluorescence data, we recorded transmission and fluorescence images simultaneously on two cameras and overlaid both datasets on the monitor with transmission signal in grey and fluorescence signal in green. For acquisitions of 3D datasets, the sample was moved through the light sheet and data was acquired plane-wise. To visualise these 3D-stacks, we rendered a 2D projection with depth colour coding. To ease orientation, a small schematic of the sample and the light sheet was displayed on screen, indicating their current orientation and position.

### Autonomous error handling

When a microscope fails during normal operation in the lab, the error is handled either by user intervention (e.g. rebooting, replugging) or a service technician. To minimize eduSPIM’s downtime and maintenance efforts even if individual hardware components failed, we implemented a multi-step error handling strategy described in detail in [9]. In brief, the computer was set up to shut down every night and reboot in the morning to reset the system to a clean state. The main application was written in Java and called from a Windows batch script that handled hardware errors by autonomously re-initializing the hardware and restarting the main application. To minimize maintenance efforts further, we also used email notifications, remote access and a fall-back mode simulating microscope operation until the error was resolved on-site.

### Clean optics design within limited space

The optics of a scientific microscope is designed to maximize imaging performance. Access is restricted to trained users for potentially dangerous setups, e.g. open beampaths. In contrast, we wanted eduSPIM be accessible to anyone interested without special training. Also, space in the museum was limited to a 60cm × 60cm × 20cm box lowered into a wooden table. We designed eduSPIM such that all optical components fit into the visible space and covered the box with a glass plate secured with a laser safety interlock. If the cover is opened, the laser shuts down until both the cover is closed and the software rebooted.

**Fig 1:**
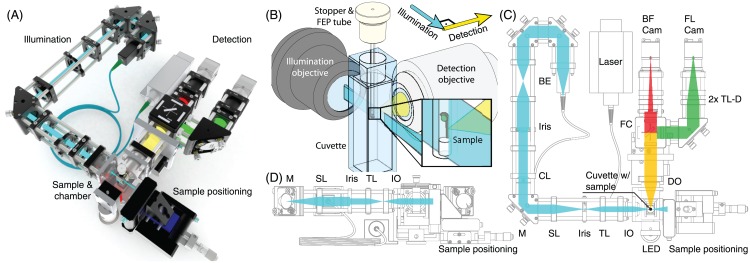
Microscope setup, light sheet geometry and beam path. (A): Computer rendering of the setup. (B): Light sheet geometry. The water-filled cuvette with the sample embedded in an FEP tube was positioned at the intersection of the illumination and detection objective’s focal planes. The sample was moved through the thin sheet of light illuminating the focal plane of the detection objective. (C): Top view of the beam path. The illumination laser was fibre-coupled into the beam expander (BE) and the light sheet formed with a cylindrical lens (CE). A mirror (M) was used to adjust the light sheet position. The light sheet was projected into the sample with an assembly of scan lens (SL), tube lens (TL) and illumination objective (IO). A red LED (LED) was used for transmission imaging. Both, fluorescence and transmission signal, were collected with the detection objective (DO), separated with a filter cube (FC) and imaged through two identical tube lenses (TL-D) simultaneously onto two cameras (BF cam, FL cam). To acquire 3D data, the imaging chamber was moved in *z* and the *z*-position of the detection objective was adjusted using motorized stages. (D): Side view of the beam path. Side and top view only differ after the illumination light passed the cylindrical lens; only this part of the beam path is illustrated.

We decided on the “classical” SPIM layout consisting of one illumination and one detection path mounted horizontally on the table (Fig 1). The sample was inserted from the top, perpendicular to either optical arm. To highlight the beam paths and guide the eye, we did not use posts, but mounted all optical components in a cage system, which was also expected to provide better long-term stability. We used a cylindrical lens to create a static light sheet [3] instead of a scanned light sheet [10], thereby avoiding a moving, potentially error-prone, scan mirror.

### Closed sample chamber

**Fig 2:**
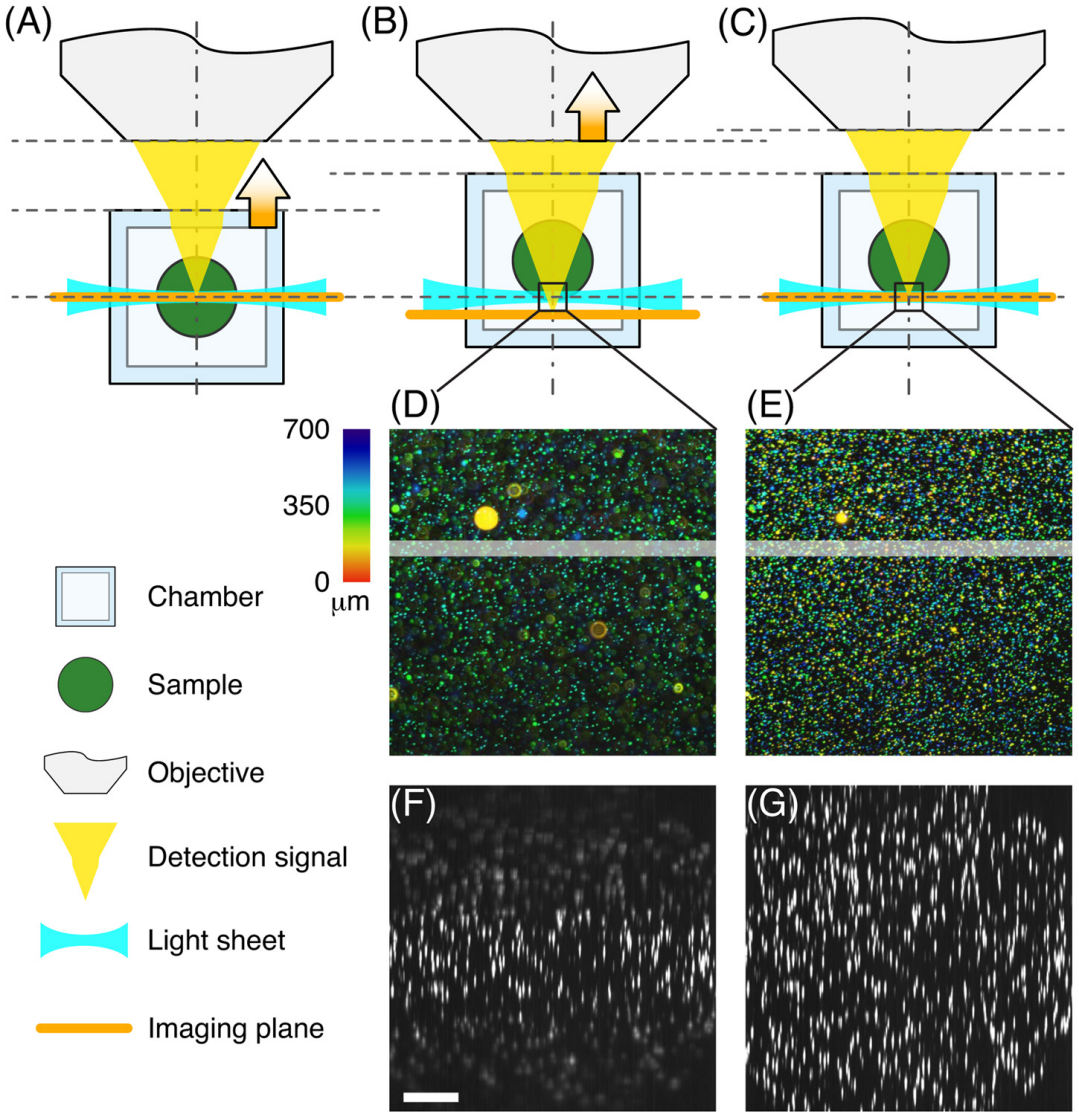
Adjustment of the detection objective position. (A): During alignment, the imaging plane and light sheet were superpositioned in the centre of the sample. (B): A 3D image stack was acquired by moving the medium-filled chamber containing the sample through the light sheet, but imaging plane and light sheet did not overlap anymore due to refractive index mismatch between medium and air. (C): The detection objective was moved to compensate for the mismatch. (D): Colour-coded projection of ø500nm fluorescent beads acquired without moving the detection objective. Beads towards both ends of the stack were out-of-focus. (E): Same stack acquired with refocusing of the detection objective. All illuminated beads were in-focus. (F): The stack shown in (D) was resliced and planes 350-399 were maximum intensity projected (highlighted region in (D)), showing out-of-focus beads towards the end of the stack. (G): Reslice and maximum intensity projection of the highlighted region in (E). The whole stack is in focus. Scalebar 100 m.

Classically, the medium-filled sample chamber in SPIM has an open top and clearances sealed with O-rings for the water-dipping illumination and detection objectives. The sample is inserted from the top into the refractive index matched medium and stacks are acquired by moving the sample, while light sheet and chamber remain static. After an experiment, the sample and medium are removed and the chamber is rinsed with water. While this design necessitates only one moving component—the sample—the open chamber is inadequate for long-term usage in the museum. Primarily to prevent evaporation of the immersion media, we adapted a standard fluorescence cuvette with four polished sides and a tight stopper as sample chamber. We used long working distance air objectives for illumination and detection instead of water dipping objectives. As a result the entire chamber, not only the sample, was translated during stack acquisition. Due to the refractive index mismatch between air and medium, the position of the imaged plane shifted during stack acquisition and was corrected for by moving the detection objective (Fig 2).

### Small non-optical hardware components

To ensure that visitors concentrate on the optical design, all non-optical components, i.e. computer, hardware controllers and cables, were hidden beneath a wooden cover. Since the available out-of-view space was also limited, we selected a mini PC to run the software. Normally in the lab, cameras and motors would be connected to a large tower PC via PCIe slots that were not available on the mini PC. Instead, all hardware components were chosen to connect via USB: The small CMOS cameras were powered and communicated via USB3.0. The motors were driven by controllers that, in turn, connected via USB. The control software for the push buttons was implemented on an Arduino board, which again interfaced via USB port with the mini PC.

### Instructive sample choice

For the museum, an eye-catching sample was needed that helped to visualise the principles of light sheet microscopy. We chose zebrafish embryos, because they are an important model organism not only in our research but also in fields as diverse as developmental biology and toxicology [11]. Zebrafish embryos are perfectly suited for light sheet microscopy thanks to their translucency and size of about 0.5mm × 0.5mm × 10mm. Transgenic lines are readily available. Due to legal and physiological restrictions, we decided against living transgenic zebrafish embryos in the museum but instead used fixed embryos according to the protocol described below.

**Fig 3:**
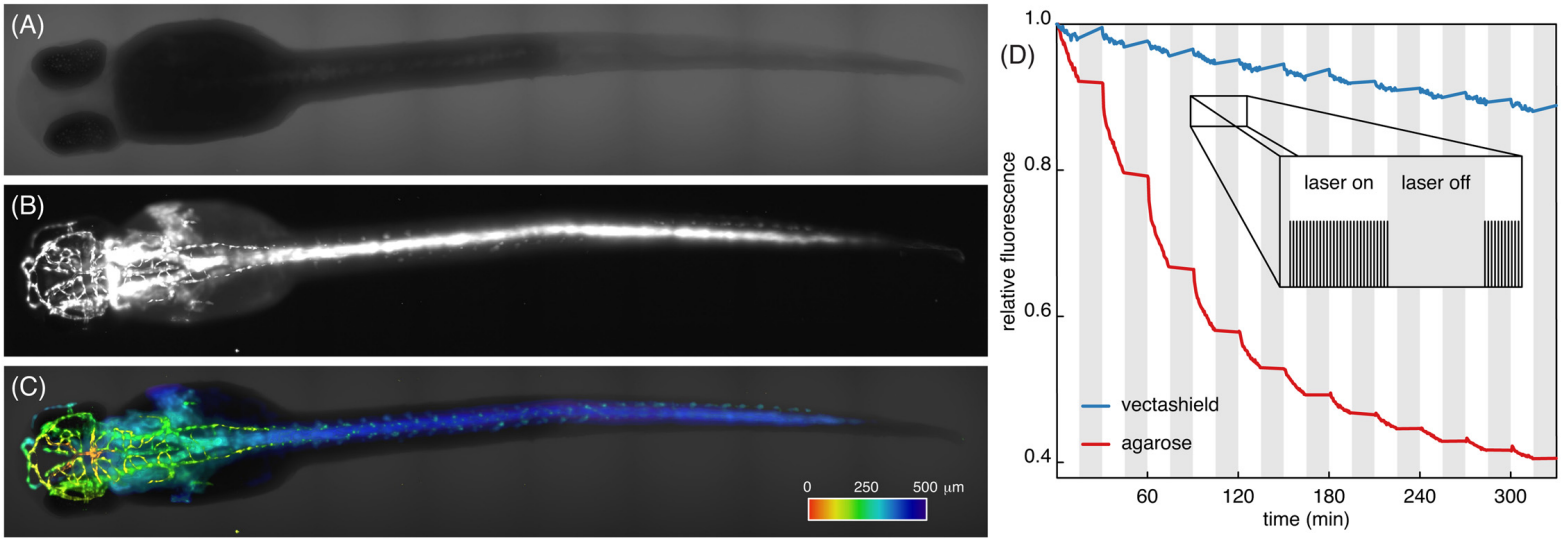
72 hpf zebrafish embryos expressing GFP in the vascular system. Prior to imaging, all zebrafish embryos were fixed and their fluorescence signal was recovered using booster-GFP. (A): Transmission image and (B): fluorescence image of vasculature of the same embryo. (C): Overlay of fluorescence and transmission data. Fluorescence data was 3D-rendered and colour coded for depth. Dataset of 75planes, colour bar, 500 m long. (D): Fluorescence signal of the sample embedded in vectashield (blue) and agarose (red). The sample was illuminated every 30s for 15min, followed by a 15min dark period (inset). Photobleaching dominated during bright periods. For mounting in vectashield, overall photobleaching was greatly reduced and even negligible during dark periods.

Crucially, we needed a fluorescent marker labelling only a subset of cells that contrasted with the transmission image, but still spanned the entire sample. At the same time, the labelled structures should be comprehensible to laymen and convey information not seen in the brightfield data. We decided for a transgenic line labelling the vascular system in the entire fish with GFP (Fig 3). As the sample is translated through the light sheet, ever-changing cross sections of the vascular network are illuminated, thus illustrating the optical sectioning of light sheet microscopes.

## Specifications of the microscope setup

A fluorescence cuvette (3.5ml, 1cm thickness, four polished sides) with a tight stopper was adapted as a sample chamber to support mounted zebrafish embryos. Using custom-built adaptors, the cuvette-chamber was mounted on a sample positioning system consisting of a manual *x*-stage (along illumination axis) and motorized *y*- (vertical) and *z*-stages (along detection axis, for stack recording). With these stages, cuvette and sample were positioned at the intersection of the illumination and detection objectives’ focal planes.

For fluorescence imaging, a 100mW, 488nm laser was coupled into the illumination arm using a fibre and a dedicated collimator producing a beam of 8mm diameter and two alignment mirrors (Fig 1C and 1D). A Keplerian 1:1 telescope (30mm focal length, BE) was used to adjust the collimation more precisely. An iris in the beam path was used to crop the beam and adjust the light sheet thickness. A horizontal light sheet was formed with a cylindrical lens (50mm focal length, CL) and projected with a second Keplerian telescope (50 and 30mm focal length, SL & TL) into the back focal plane of the air illumination objective (5x/0.1, IO) to create the vertical light sheet in the sample chamber. A mirror located between cylindrical lens and second telescope in the conjugate plane of the BFP of the illumination objective was used to shift the light sheet position along the detection axis and to focus the light sheet image onto the camera chip.

The detection objective (20x/0.40, DO) was placed on a motorized stage and was moved to correct for its shifting focal plane when the sample chamber was translated (Fig 2). When comparing the optical path lengths, one finds a correction factor of

$$\frac{d_{\text{objective}}}{d_{\text{chamber}}}=1-\frac{n_{\text{air}}}{n_{\text{water}}}\approx 0.248,$$ (1)

where *d*_chamber_ and *d*_objective_ are the distances that chamber and objective, were moved and *n*_air_ and *n*_water_ are the refractive indices of air and water. We acquired a *z*-stack in a sample of ø500nm fluorescent microspheres (Estapor, Merck, Darmstadt, Germany) embedded in a column of 1.5% low melting agarose (LMA, Sigma-Aldrich Chemie GmbH, Taufkirchen, Germany). By manually adjusting the detection objective’s positions for different *z*-positions, we measured a correction factor of 0.246, in agreement with the theoretical value. We found that any spherical aberrations introduced by refractive index mismatch can be considered negligible for our low NA detection objective.

The selected detection objective provided a field number of 20mm, thus overfilling the fairly small camera chips (6.8mm × 5.4mm, 8.7mm diagonal). In order to shrink the image to the size of the camera chip, we used a tubelens (TL-D) of 75mm focal length (instead of 165mm common for Zeiss). With this modified tubelens, our system had an overall magnification of 9.1 and a field number of 9.1mm. At this magnification, the image of the zebrafish embryo filled the whole width of the camera chip. With our detection design, we benefited from the larger numerical aperture of the 20× lens as compared to a 10× objective.

For transmission imaging, a red (635nm) LED was placed behind the imaging chamber. Both, fluorescence and transmission signals were acquired simultaneously, split by a dichroic mirror (594nm), a GFP bandpass filter (550/88nm) and a long pass filter (594nm), all mounted in a filter cube (FC), and imaged onto two CMOS cameras (BF Cam and FL Cam).

Illustrated step-by-step instructions to built an eduSPIM and a list of parts including manufacturer information and part numbers are provided in S1 Text. A 3D computer model of the whole eduSPIM assembly is provided in S1 3DModels.

## Preparation of durable, fluorescent zebrafish embryos

Light sheet microscopes are mainly used to image dynamic processes in living samples. Such experiments are usually limited to a few hours or days and the protocols for sample embedding are optimized to interfere as little as possible with developmental processes, such as growth or gas exchange. In the museum, the experiment duration is essentially unlimited: Visitors may be acquiring images during the whole day and the sequence of illuminated regions is unpredictable. Ideally, the sample should last for the duration of the exhibition. In reality, the fixed sample is affected severely by photobleaching: In contrast to a living sample, it does not re-synthesize fluorescent molecules. In extreme cases, the sample might even decay. Luckily, restrictions on embedding protocol and medium are less stringent for a fixed sample than for a living sample. The medium may even be toxic as long as it minimizes photobleaching.

### Minimize photobleaching with antifade

In order to estimate and optimize the lifetime of the sample, we acquired *z*-stacks repeatedly and measured the average fluorescence signal (Fig 3D). We performed the experiments for samples mounted in both 3% LMA and vectashield (BIOZOL Diagnostica Vertrieb GmbH, Eching, Germany). For mounting in LMA, photobleaching rendered even a non-illuminated sample unusable within a day. Apparently, fluorescent molecules also decayed when the fixed sample was stored at room temperature (exceeding 30°C in summer) and not in the fridge. For embedding with vectashield, more than 85% of fluorescence intensity remained after the acquisition of 300 stacks and the decay of fluorophores at room temperature was negligible. Since, on average, 50 stacks were acquired per day and only part of the sample was illuminated for each stack acquisition, we exchanged the sample every two to three weeks.

### Sample mounting protocol

All animals were treated in accordance with EU directive 2011/63/EU as well as the German Animal Welfare Act. Zebrafish (*Danio rerio*) adults and embryos were kept at 28°C and were handled according to established protocols [12]. Zebrafish of the *casper*-strain (lacking pigmentation [13]) expressing GFP in the vasculature (*Tg(kdrl:EGFP)* [14]), were crossed and the offspring raised in E3 [12]. At 24 hours post fertilization (hpf), the embryos were treated with 0.2 M 1-phenyl 2-thiourea (PTU, Sigma-Aldrich) to inhibit melanogenesis especially in the eyes.

After hatching, usually between 48 and 72 hpf, zebrafish embryos were anaesthetized with 130mg 1^−1^ Tricaine (Sigma-Aldrich) and fixed for 4h at room temperature (RT) using 4% paraformaldehyde (Sigma-Aldrich) in PBS. To permeabilize fixed embryos, we washed 2× with 0.3% Triton-X (Serva Electrophoresis GmbH, Heidelberg, Germany) in PBS, rinsed with water, incubated in icecold acetone for 8min, rinsed with water and washed with 0.3% PBS-Triton-X again. In order to recover fluorescence signal lost during fixation, embryos were blocked for 2h at RT using 10% normal goat serum (NGS, Jackson Immuno Research Laboratories, Inc., Suffolk, UK) in PBS-Triton-X. Afterwards, they were incubated overnight at 4°C using 1:400 GFP-Booster (ChromoTek GmbH, Planegg-Martinsried, Germany) in PBS-Triton-X-NGS and washed 2× with 0.3% PBS-Triton-X. Then, embryos were transferred into PBS and stored at 4°C. Fresh samples were prepared every two months.

For imaging, zebrafish embryos were embedded according to a modified protocol from Kaufmann et al. [15] either in 3% LMA or in vectashield in a 3cm long piece of fluorinated ethylene propylene (FEP) tube with inner diameter of 0.8mm and outer diameter of 1.6mm (S 1815-04, BOLA, Grünsfeld, Germany). FEP has a refractive index of 1.338, which is sufficiently close to the refractive index of water that optical aberrations are negligible. To prevent the embedding medium from sliding from the tube (caused by high ambient temperatures in the museum), both ends were closed with nail polish. The FEP tube was inserted into a small hole drilled into the cuvette stopper and the PBS-filled cuvette mounted on the motion control assembly. The zebrafish embryo was always imaged dorsally to ensure that the visibility of blood vessels was not obstructed by the scattering yolk sac.

## Educational and outreach activities

**Fig 4:**
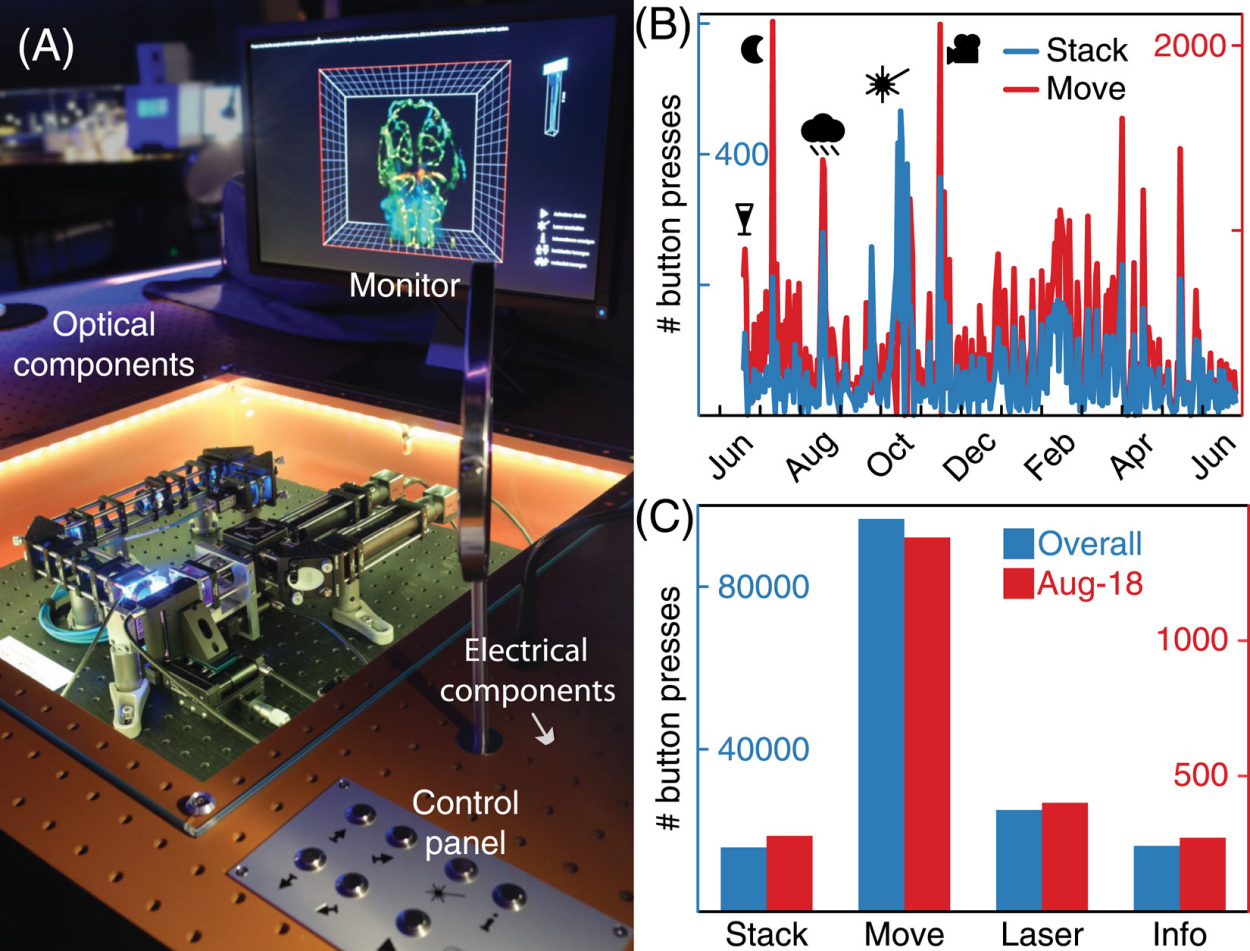
eduSPIM in the museum. (A): Photograph of the setup combined from two individual photographs acquired with different polarization. All optical components were placed beneath a glass cover, which also included a laser safety interlock. In order to keep the appearance of the system as clean and clear as possible, all controllers, the Arduino board and the PC were hidden below the table. The system was operated via a control panel including seven push buttons while the sample was viewed on a monitor. (B): Usage statistics: stacks (blue) and sample moves (red). Peaks in number of acquired stacks correspond to the official opening day of the exhibition (champagne glass), the long night of open museum (moon), a stretch of extremely rainy days in Dresden (cloud), the photobleaching experiments we conducted (laser, see Fig 3) and a media festival (camera). (C): Distribution of button presses overall (blue) and on Aug 18, 2015 (red), automated photobleaching experiments were not counted towards the overall number of button presses.

In addition to designing and building a light sheet microscope for the museum (Fig 4A), we developed and programmed a suite of tools to aid in understanding the exhibit, to estimate and maximize eduSPIM’s reach and to ensure consistent imaging quality.

### Display background information

The whole eduSPIM concept was designed specifically towards easy operation, clarity of the optical principles and descriptiveness of the sample. To still provide background information to interested visitors, we included a seventh push button in the operational panel, which was used to bring up additional information on screen such as a blueprint of the system annotated with beampath and key optical components (comparable to the illustration shown in Fig 1C) and a schematic of the light sheet geometry within the context of the actual setup. Furthermore, we provided background information on zebrafish as model organism and displayed a QR-code pointing to the eduSPIM website.

### eduSPIM usage statistics, live view and website

The last image acquired on eduSPIM (“live view”) and summary of daily button presses were uploaded into a cloud storage. We used this information in two ways: Firstly, to identify any problems such as sample bleaching or misalignment and to determine if, and how often, the sample needed to be replaced. Secondly, to extract usage statistics (Fig 4B and 4C): how much impact our microscope had, how many visitors were reached and how they operated eduSPIM. For example, we easily identify key events in the statistics such as the long night of open museum.

We registered the URL eduspim.org to reach potential visitors before and after their stay in the museum. On the website, we present a shortened version of the information page in the museum. Additionally, we uploaded photographs of the setup, how it was first built in the lab and then integrated into the exhibition. From eduspim.org, we also link to an “eduSPIM live view” where the live view and statistics information are presented in a condensed and understandable way and prepared for display on public screens, e.g. in the museum’s cafeteria.

## Discussion and outlook

In this manuscript, we presented an educational light sheet microscope and discussed the key concepts of hardware design and sample preparation that are specifically tailored to attract visitors with a non-scientific background. We reduced the control panel of the microscope to the essentials and created intuitive pictograms labelling the push buttons. The layout of eduSPIM’s optics fulfilled two central requirements: Long-term stability and clarity such that the form of the microscope conveyed its function. We chose zebrafish embryos as an eyecatching sample with a fluorescent vasculature marker that was comprehensible to laymen. We optimized the sample preparation to minimize photobleaching and prolong the sample’s durability. Although we have developed the concepts discussed here specifically for the eduSPIM, they are also valid for other educational (microscopy) projects.

Our eduSPIM is exhibited at the special exhibition “Hi Lights!” in the Technische Sammlungen Dresden, where it will remain for one year. At the time of writing, more than 18000 stacks have been acquired and the microscope has been continuously running since the exhibition had opened more than nine months ago. While our microscope was designed specifically for this exhibition, its life span is not limited by the exhibition’s duration. Instead, we are currently exploring whether eduSPIM could be hosted by additional museums in the years to come. Moreover, there are a multitude of outreach activities on the Dresden campus, including the long night of science (once a year on a Friday night, research institutes open their doors to the public), the “science goes to school” project (international PhD students visit Dresden schools to convey an understanding of science to the students) and Juniordoktor (students aged 8–18 take courses at Dresden research institutes and receive a “doctoral degree”). eduSPIM may become a valuable complement for any of them.

Moreover, eduSPIM was designed to be a fully functional microscope. Therefore, it can be used not only for teaching purposes, but also adapted for research groups looking for an easy-to-operate light sheet microscope. For scientific imaging tasks, it may be desirable to include rotation of the sample for optimal positioning and multiview acquisition. While this feature is currently lacking in eduSPIM, it can be easily incorporated by modifying the sample mounting presented by Bruns et al. [16]: If the FEP tube was pulled through a sealed hole in the cuvette stopper, it could easily be attached to a rotational stage. Then, the tube (containing the sample) may be rotated within the chamber.

Finally, the design presented here can also serve as a template to develop an educational kit, which could be used in schools and universities. In practical sessions taught in our lab, even untrained users are able to build light sheet microscopes within a day. This time frame agrees well with the time usually available for practical courses. A light sheet microscopy kit may extend the feasibility of such a practical beyond specialist labs. Ideally, with such a kit, light sheet microscopy may be taught in undergrad courses or even to high school students.

## Supporting Information

S1 Text Instructions for building an eduSPIM. This supplementary file provides the information needed for building an eduSPIM by giving illustrated, step-by-step instructions and providing a detailed list of parts.
(PDF)

S1 3DModels Computer model of the whole eduSPIM setup. Complete, assembled eduSPIM is provided as .step file. The assembly also contains the models for all homebuilt parts.
(ZIP)
