# Supplementary material for: eduSPIM: Light Sheet Microscopy in the Museum
Source: PLoS One. 2016 Aug 25;11(8):e0161402. doi: 10.1371/journal.pone.0161402 (PMC4999180; doi:10.1371/journal.pone.0161402)
Supplement: S1 Text — This supplementary file provides the information needed for building an eduSPIM by giving illustrated, step-by-step instructions and providing a detailed list of parts. (PDF) [file pone.0161402.s001.pdf]

S1 Text. Instructions for building an eduSPIM

Wiebke Jahr[1,*], Benjamin Schmid[1], Michael Weber[1], Jan Huisken[1,*]

1 Max Planck Institute of Molecular Cell Biology and Genetics, 01307 Dresden, Germany
* jahr@mpi-cbg.de, huisken@mpi-cbg.de

Instructions for building an eduSPIM

This supplementary file provides the information needed for building an eduSPIM using the Thorlabs 30 mm cage system and optics with a diameter of 1 inch. For the sake of this instruction, we consider three main assembly groups: (i) the illumination arm, (ii) the detection arm and (iii) the sample control unit (stage). Illustrated, step-by-step instructions for each group are given in supplementary Figures 1 – 5. For each step, a schematic of the setup including the beam path is shown on the left and a to-scale line drawing of the actual components is shown on the right. A detailed list of parts is given in Table 1. The *.step*, *.igs* and *.sldprt* files for the whole system are provided in additional supplements and include the information for all homebuilt adaptors and the cuvette mounting stage.

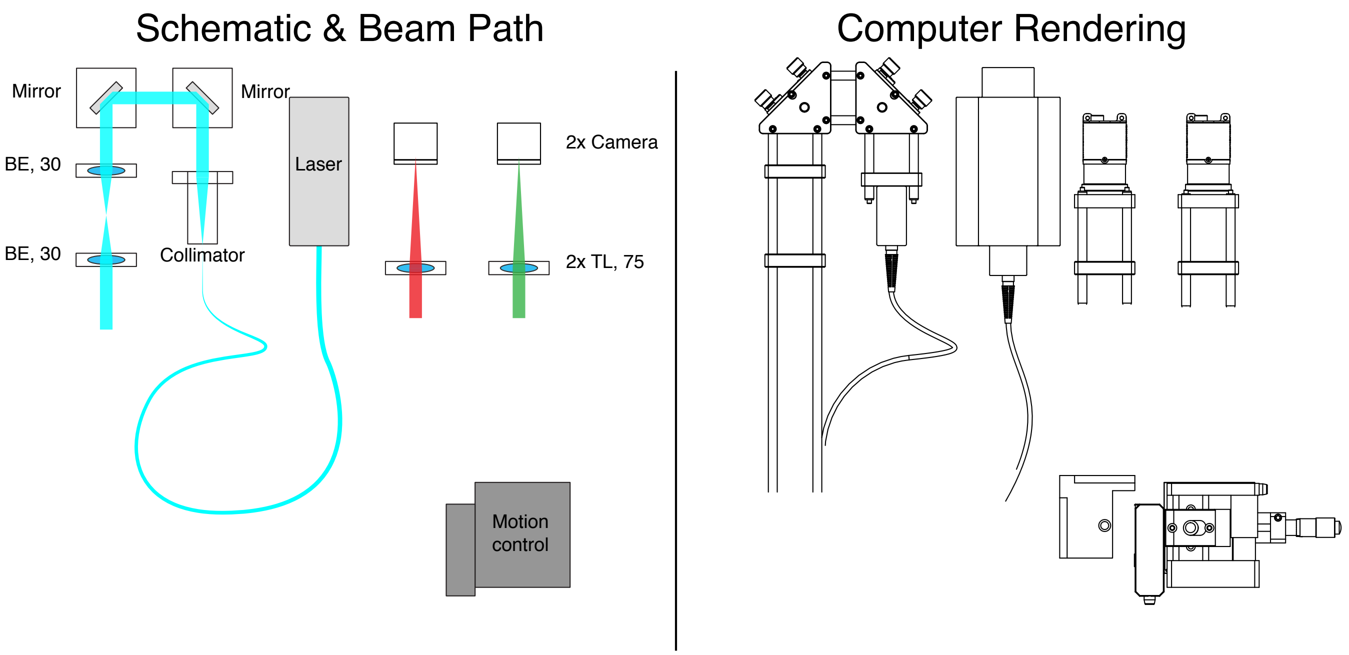

Figure 1.

- General
  - When assembling the cage system, use some additional cage plates with large aperture (CP03/M) to stabilize the cage. Use these plates to elevate the whole cage structure on 50 mm posts.
  - Assemble the homebuilt corner piece that will be used later to connect illumination and detection arm and mount it on a 25 mm post (only shown in the computer rendering).
- Illumination
  - Mount the laser on the breadboard and place the fibre in the collimator.
  - Place the collimator in the customized cage plate and adjust laser collimation.
  - Mount the two alignment mirrors and align the laser beam to go through the centre of the cage system.
  - Mount the two lenses of the telescope (BE, both 30 mm). In principle, the telescope could be used to change the beam diameter (magnifying or demagnifying). Here, the 1:1 telescope is just used to collimate the beam more precisely.
- Detection
  - Mount the cameras in the cage system using C-mount SM-1 adaptors and SM-1 tubes.
  - Mount the tube lenses (TL, 75 mm).
  - Adjust the camera/tube lens distance by imaging an “infinitely” far away object (e.g. a structure outside the window) onto the camera chip. For the infinity corrected detection objective, this is the correct distance.
- Stage
  - mount the manual linear stage (for adjusting x-position of the sample) on the breadboard.
  - mount the two motorized stages (for z- and y-direction) on the manual stage.

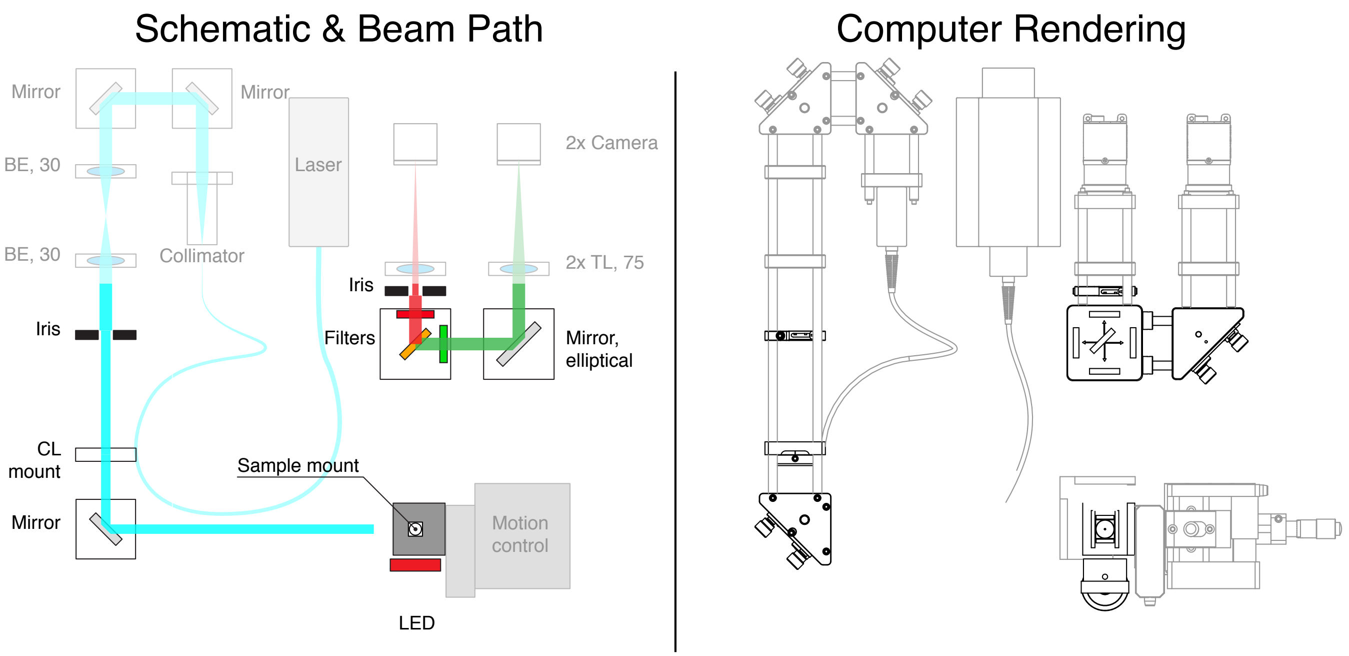

Figure 2.

- Illumination
  - Mount the iris and the empty rotational mount for the cylindrical lens (CL mount) in the cage system. The iris can be used to adjust the light sheet thickness by cropping the beam. The cylindrical lens will be placed later.
  - Mount the mirror and align the beam to go through the centre of the cage system. The mirror will be used later to focus the light sheet image onto the camera chip.
- Detection
  - Mount the elliptical mirror into the cage for the fluorescence camera.
  - Mount the iris onto the cage for the transmission camera. It is used to increase the depth of field of the transmission image.
  - Mount the filter cube and connect the cage system of the transmission and fluorescence detection arms.
- Stage
  - Mount the adaptors for holding the cuvette onto the motorized stages and place the cuvette containing a sample.
  - Mount the red transmission LED on the breadboard.

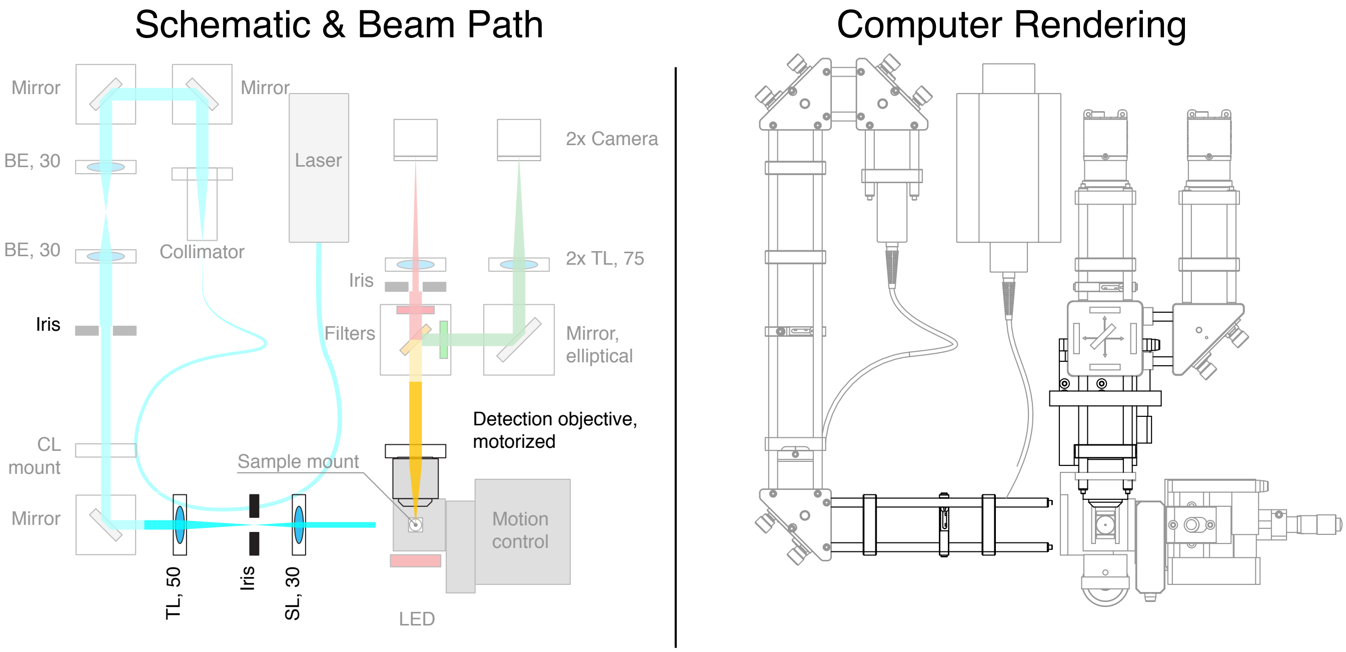

Figure 3.

- Illumination
  - Place the tube lens (TL, 50 mm) in the cage system and adjust it until the mirror is in its back focal plane.
  - Mount the iris in the focal plane of the tube lens. The iris will be used to crop the light sheet height to the size of the camera chip and to prevent bleaching of sample regions that are not imaged.
  - Place the scan lens (SL, 30 mm) and align until the beam is collimated.
- Detection
  - Place the motorized detection objective in the cage system and slide the detection arm into the home built corner piece connecting the illumination and detection arm.
  - Using transmission light, adjust the distance of the whole detection arm to the cuvette until you get a sharp image of the centre of the sample.

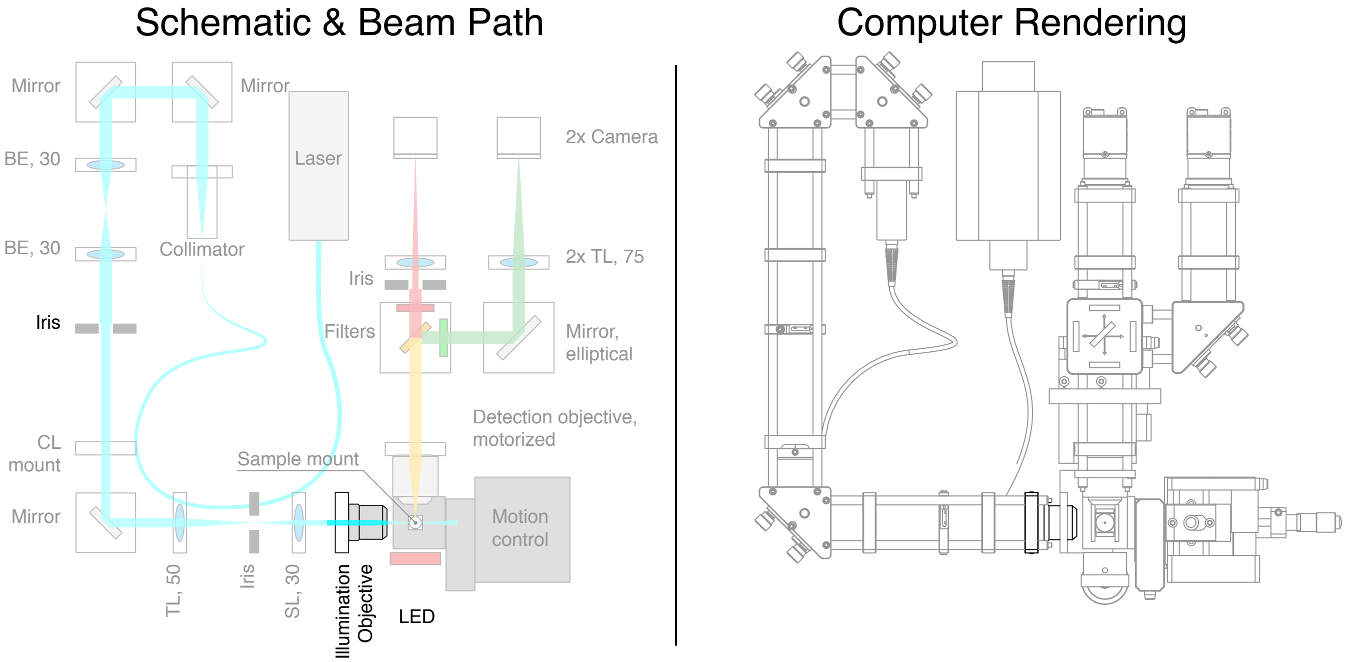

Figure 4.

- Illumination
  - Place the illumination objective such that its back focal plane is in the focal plane of the scan lens.
  - Slide the illumination arm into the corner connecting both arms of the SPIM.
- Align illumination and detection
  - Place a water-filled cuvette in the sample mount and remove the filters from the filter cube to image the scattered signal of the illumination beam.
  - Move the whole illumination arm until the waist of the Gaussian beam is in the centre of the camera's field of view. Make sure that the illumination arm is not tilted.
  - Adjust the alignment mirror to focus the image of the Gaussian beam onto the camera.

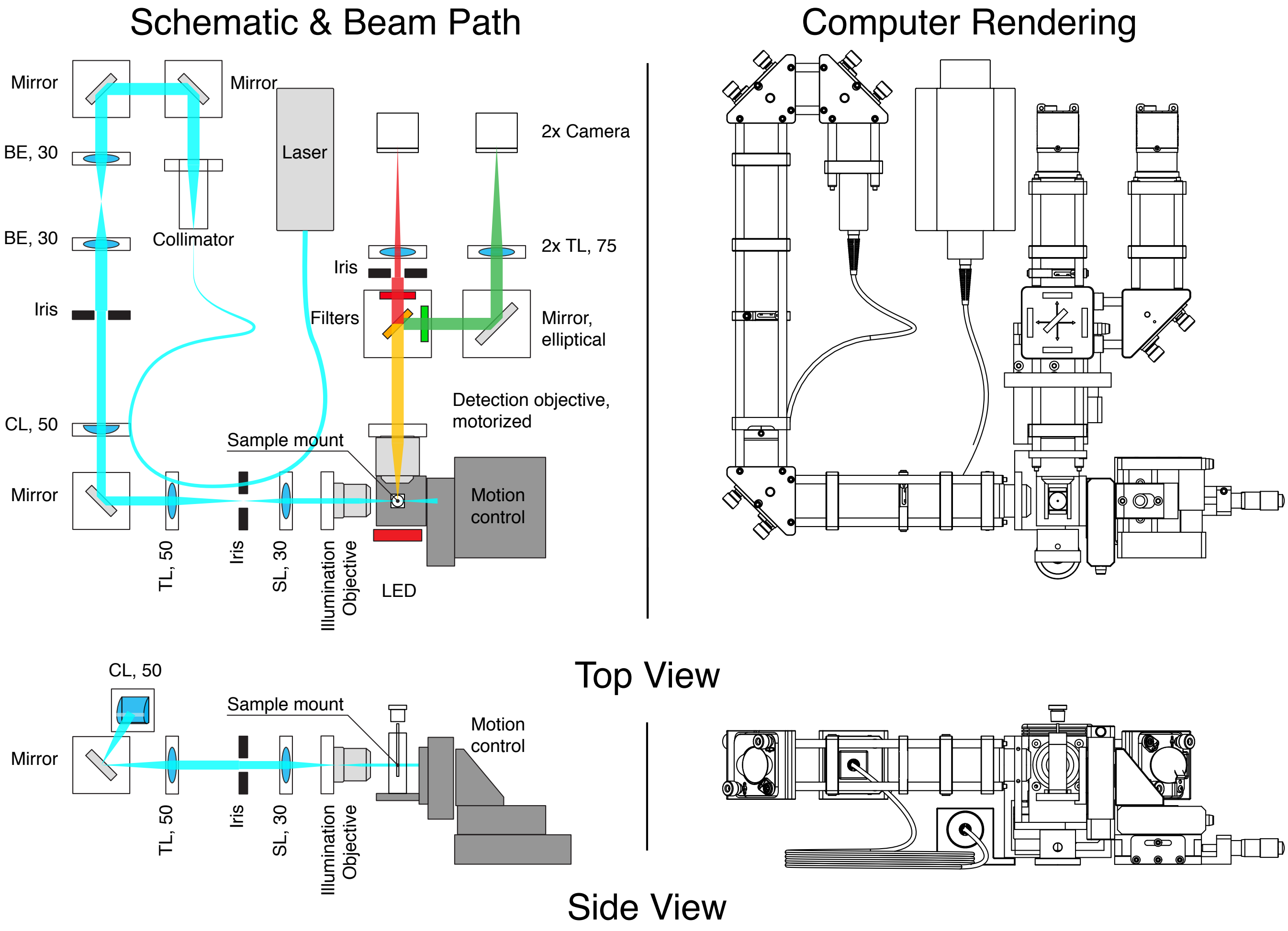

Figure 5.

- Create the light sheet
  - Place the cylindrical lens in its mount and move the mount until you get a sharp line on the mirror. Rotate the mount until the line is horizontal.
  - Additionally to the top view, a side view is shown in Figure 5.
- Fine-tune the alignment
  - Place the filters back in the filter cube and a sample of fluorescent beads in the cuvette.
  - Focus by adjusting the mirror.
  - Rotate the cylindrical lens to adjust a tilted light sheet.
  - If the light sheet is tilted in x-direction, the illumination and/or the detection arm are tilted.
  - Close the iris to crop the light sheet to the desired height.
  - Place the actual sample in the cuvette. Focus in the centre of the sample by adjusting the mirror.
  - Move the transmission camera to ensure that the focus of the transmission and the fluorescence image overlap and close the iris to get the desired depth of field.
- Calibrate the refocusing
  - Move to the front of the sample using the motorized z-stage. Adjust the focus by moving the motorized detection objective. Note the positions of both stages.
  - Move to the back of the sample and adjust the focus and note the positions of both stages.
  - The calibration curve for refocusing is acquired by linear interpolation between these two values.

Table 1. List of parts. Companies: Toptica (Toptica Photonics AG, Germany), Stemmer (Stemmer Imaging GmbH, Germany), Thorlabs (Thorlabs Inc, New Jersey, USA), Zeiss (Carl Zeiss AG, Germany), Chroma (Chroma Technology Corporation, Vermont, USA), Semrock (Semrock, Inc, New York, USA), PI (Physik Instrumente GmbH & Co. KG, Germany), Arduino (Arduino, Italy), APEM (APEM GmbH, Germany)

	Part	Specifications	Part No.	Manufacturer	Amount
Light sources	Laser	100 mW, 488 nm	iBeam smart 488	Toptica	1
	Collimator	8 mm diameter	Fibreout	Toptica	1
	LED	635 nm	CCS TH-27/27-RD	Stemmer	1
	Power supply	for LED	CCS PSB-1024V-WW	Stemmer	1
Illumination optics	Alignment mirror	25.4 mm, 400 to 750 nm	BB1-E02	Thorlabs	2
	Lens	30 mm, 350 to 700 nm	LB1757-A	Thorlabs	1
	Lens	30 mm, 350 to 700 nm	LB1757-A	Thorlabs	1
	Cylindrical lens	50 mm, 350 to 700 nm	LJ1695RM-A	Thorlabs	1
	Lightsheet mirror	25.4 mm, 400 to 750 nm	BB1-E02	Thorlabs	1
	Scan lens	50 mm, 350 to 700 nm	LB1471-A	Thorlabs	1
	Tube lens	30 mm, 350 to 700 nm	LB1757-A	Thorlabs	1
	Illumination objective	WD 4.4 mm	LSFM 5x/0.1	Zeiss	1
Detection optics	Detection objective	WD 7.3 mm	LD Epiplan 20x/0,40	Zeiss	1
	Dichroic	594 nm	zt 594 RDC	Chroma	1
	GFP bandpass	550/88 nm	550/88 BrightLine HC	Semrock	1
	Transmission longpass	594 nm	594 LP Edge Basic	Semrock	1
	Alignment mirror	elliptic, 400 to 750 nm	BBE1-E02	Thorlabs	1
	Tube lens	75 mm, 350 to 700 nm	LB1901-A	Thorlabs	2
	Camera	CMOS 1280x1024 px	DCC3240M	Thorlabs	2
Motion control	x-stage	13 mm range	MT1/M	Thorlabs	1
	y-motor	25 mm range	M112.1DG	PI	1
	z-motor	15 mm range	M111.1DG	PI	1
	Objective motor	15 mm range	M111.1DG	PI	1
Opto-mechanics	Cuvette	3500 µl, fluorescence	CV10Q3500FS	Thorlabs	1
	Breadboard	600 mm × 600 mm	MB6060/M	Thorlabs	1
	Rod	10 in, 6 mm	ER10	Thorlabs	4
		6 in, 6 mm	ER6	Thorlabs	4
		3 in, 6 mm	ER3	Thorlabs	12
		2 in, 6 mm	ER2	Thorlabs	4
		1 in, 6 mm	ER1	Thorlabs	4
		0.5 in, 6 mm	ER05	Thorlabs	4
	Post	50 mm	RS2P4M	Thorlabs	7
	Post	25 mm	RS1P4M	Thorlabs	1
	Clamp		CF125C/M	Thorlabs	8
	Cage plate	35 mm aperture	CP03/M	Thorlabs	7
	Lens mount		CP08	Thorlabs	6
	Rotational mount	for cylindrical lens	CRM1L/M	Thorlabs	1
	Mirror mount	round	KCB1C/M	Thorlabs	3
	Mirror mount	elliptic	KCB1E/M	Thorlabs	1
	Filter cube		DFM/M	Thorlabs	1
	Camera mount	adaptor	SM1A9	Thorlabs	2
		lens tube	SM1L20	Thorlabs	2
		cage plate	CP12/M	Thorlabs	2
	Blank mount	for collimator & objectives	CP01/M	Thorlabs	3
	Iris		CP20S	Thorlabs	3
Control	PC		NUC5i5RYK	Intel	1
	Arduino		Arduino MEGA 2560	Arduino	1
	Push button		AV0630C900	APEM	7
Custom made parts	Adaptors to hold the cuvette				1
	Adaptors to mount the motorized detection objective				1
	Corner pieces to connect illumination and detection arm				1
	Baseplate for push buttons				1
